# School and household tuberculosis contact investigations in Swaziland: Active TB case finding in a high HIV/TB burden setting

**DOI:** 10.1371/journal.pone.0178873

**Published:** 2017-06-05

**Authors:** Piluca Alonzo Ustero, Alexander W. Kay, Katherine Ngo, Rachel Golin, Bhekisisa Tsabedze, Bulisile Mzileni, Jessica Glickman, Mildred Wisile Xaba, Gcinile Mavimbela, Anna Maria Mandalakas

**Affiliations:** 1 The Global Tuberculosis Program, Texas Children's Hospital, Section of Global and Immigrant Health, Department of Pediatrics, Baylor College of Medicine, Houston, Texas, United States America; 2 Baylor College of Medicine Children's Foundation - Swaziland, Mbabane, Swaziland; 3 Swaziland National School Health Program, Mbabane, Swaziland; 4 Swaziland National Tuberculosis Control Program, Mbabane, Swaziland; National Institute of Health, ITALY

## Abstract

**Background:**

Investigation of household contacts exposed to infectious tuberculosis (TB) is widely recommended by international guidelines to identify secondary cases of TB and limit spread. There is little data to guide the use of contact investigations outside of the household, despite strong evidence that most TB infections occur outside of the home in TB high burden settings. In older adolescents, the majority of infections are estimated to occur in school. Therefore, as part of a project to increase active case finding in Swaziland, we performed school contact investigations following the identification of a student with infectious TB.

**Methods:**

The Butimba Project identified 7 adolescent TB index cases (age 10–20) with microbiologically confirmed disease attending 6 different schools between June 2014 and March 2015. In addition to household contact investigations, Butimba Project staff worked with the Swaziland School Health Programme (SHP) to perform school contact investigations. At 6 school TB screening events, between May and October 2015, selected students underwent voluntary TB screening and those with positive symptom screens provided sputum for TB testing.

**Results:**

Among 2015 student contacts tested, 177 (9%) screened positive for TB symptoms, 132 (75%) produced a sputum sample, of which zero tested positive for TB. Household contact investigations of the same index cases yielded 40 contacts; 24 (60%) screened positive for symptoms; 19 produced a sputum sample, of which one case was confirmed positive for TB. The odds ratio of developing TB following household vs. school contact exposure was significantly lower (OR 0.0, 95% CI 0.0 to 0.18, P = 0.02) after exposure in school.

**Conclusion:**

School-based contact investigations require further research to establish best practices in TB high burden settings. In this case, a symptom-based screening approach did not identify additional cases of tuberculosis. In comparison, household contact investigations yielded a higher percentage of contacts with positive TB screens and an additional tuberculosis case.

## Introduction

The World Health Organization recommends active case finding and contact investigation among household and close contacts of infectious TB cases in middle and high TB-burden settings[1,2]. Systematic screening of high risk groups, including close contacts to infectious TB, is part of Pillar 1 in the World Health Organization’s End TB Strategy [3]. There is robust evidence in favor of household contact investigations as a means to identify new cases and prevent morbidity through early identification[4]. However, this policy is seldom implemented in TB high burden settings, as authorities hesitate to allocate resources towards case identification and prevention, rather than case management[5].

The Stop TB Partnership and TB REACH initiative have supported the development of novel active case finding programs. A meta-analysis of such projects demonstrated that the contribution of contact investigations to overall case notifications ranged widely between < 1 and 14%[6]. Active case finding programs with less restrictive symptom criteria for screening demonstrated increased rates of case detection. However, among projects with contact investigation components, only 2 of 19 evaluated any TB contacts outside of the household[6]. Therefore, the best approach to performing contact investigations outside of the household remains relatively unknown in TB high-burden settings.

The majority of TB transmission in high-burden settings occurs outside of the home environment after early childhood. Evaluation of TB genotypes in such settings indicates that—in older children, adolescents, and adults—TB infection occurs outside of the home in 81% of cases[7]. Similarly, another study demonstrated no association between household contacts with infectious TB and evidence of TB infection in adolescents[8]. In addition, older adolescents in high HIV/TB burden settings are at increased risk for acquiring tuberculosis infection and the development of TB disease compared to children between the ages of 5 and 14[9,10]. In adolescents, as many as 50% of their tuberculosis infections are estimated to occur in the school environment[11]. However, there is very little data from TB high burden settings exploring the value of, or best approach to, performing contact investigations in schools following identification of infectious TB in a student.

Therefore, with support from the TB REACH Initiative, we initiated school-based contact investigations to complement household contact and source case investigations in Swaziland, a high TB/HIV burden setting. Here we present the experience and outcomes of this intervention.

## Materials and methods

The Baylor College of Medicine Children’s Foundation-Swaziland (BCMCF-SD) TB REACH funded “Butimba” project has previously been described elsewhere[12]. In brief, this was a project designed to improve TB case finding, through household contact investigations, and case detection through the expanded use of GeneXpert in children and adolescents living in Swaziland. In addition, as reported here, the Butimba Project also piloted school-based contact investigations. In order to do so, the BCMCF-SD Butimba Project staff partnered with the Swaziland Ministry of Health’s School Health Programme (SHP) and the Swaziland National Tuberculosis Control Programme (NTCP). Positive GeneXpert diagnosis of a student or teacher prompted a school contact investigation.

Following identification of a TB index case with a school affiliation, the Butimba Project team obtained verbal consent from the caregiver and contacted the SHP. The SHP then communicated with the school to introduce the Butimba Project opportunity and describe the purpose of TB contact investigations and TB education. If the school was amenable to the event, the project team established a date and time to provide the services.

On the day of the intervention, Butimba staff delivered a school-wide health education session. Following the education session, the Butimba staff performed a TB screening and specimen collection, if indicated. At the primary schools, all children in the TB index case’s grade and grades 5–7 were screened. At secondary schools, all children in the TB index case’s grade were screened. The Butimba staff screened additional students at primary and secondary schools outside of the specified grades at the request of a teacher or administrator. The screening results were recorded in a School Contact Tracing Tool (S1 Fig). The Butimba staff maintained confidentiality of the index case throughout the intervention. Students were asked about the presence of any one of four symptoms. In a person with recent close contact to a TB index case, a positive screen consisted of an affirmative response to any one of the following: any cough, fever, night sweats or weight loss. The Butimba staff set up an outdoor specimen collection station and attempted sputum collection from all students with a positive symptom screen for tuberculosis, after nurse or physician approval. A GeneXpert MTB/RIF (Xpert, Cepheid, USA) was performed on all sputum specimens collected as per standard of care in Swaziland. This test is recommended by the World Health Organization as the initial test in children, adolescents and people living with HIV. It is a fully automated molecular polymerase chain reaction assay designed to detect *Mycobacterium tuberculosis* and rifampin resistance, and is capable of providing results in less than 3 hours. Here it was performed at regional labs under programmatic conditions. In Swaziland, a baseline acid-fast bacilli smear (Ziehl-Neelsen stain) and liquid culture (Mycobacterium Growth Indicator Tube) are performed at the National Tuberculosis Laboratory on all persons with a positive GeneXpert result or those being initiated on TB treatment based on clinical suspicion. If a student’s symptoms merited additional evaluation, the student received a referral to the BCMCF-SD clinic or BMU where the index case was identified.

After screening, students engaged in an educational soccer-based activity known as Kick TB & HIV. This program is well established in South Africa and over 127,000 students have been reached through this initiative[13]. This was the first introduction of Kick TB & HIV in Swaziland and a description of the educational program is described in S2 Fig. Following completion of the program, the BCMCF-SD provided the school with a certificate of completion and gave students information, education, communication (IEC) school materials, as well as a soccer ball with TB prevention messaging.

The Butimba Project staff entered data into a web-based database developed and secured by the Baylor College of Medicine’s Dan L. Duncan Institute for Clinical and Translational Research Center (ICTR) (Houston, TX). The TB index cases and linked household and school contacts were described with descriptive statistics. Characteristics and outcomes of contacts following household vs. school TB exposure were compared with odds ratios using the Baptista-Pike method and significance was determined by Fisher’s exact testing. Statistical analysis was performed with Prism 7.0 (GraphPad, LaJolla, California, USA).

The Butimba project executed all clinical investigation supporting the reporting of these findings according to the principles expressed in the Declaration of Helsinki. All participants gave oral consent for participation in the Butimba program. Anonymously analysed data required no individual participant consent for program analysis. The project team received approval from all necessary ethical bodies including the Baylor College of Medicine Children’s Foundation Swaziland, the Swaziland Ethics Committee (24047712/24045469) in Swaziland, and the Baylor College of Medicine Institutional Review Board (H-35028), Houston, Texas, USA.

## Results

From June 2014 to March 2015, the Butimba project was notified of 7 GeneXpert confirmed pediatric TB index cases that were attending school (Table 1). HIV status was positive in 4 (57%) of the adolescent index cases; the median age was 13 years (range 11 to 20) and 3 (43%) were female. Cough was present in 86% of index cases, while fever, night sweats, and weight loss were identified in 2 (29%) cases. Acid-fast bacilli (AFB) smear testing was positive in 71% (5/7). Mycobacterial culture results were available for 5 cases, and all were positive including the two cases with negative AFB smears.

**Table 1:** TB index case demographic and clinical data.

TB Index Cases (n = 7)	
Age (years), median (range)	13 (11–20)
Female, n (%)	3 (43)
HIV Positive n (%)	4 (57)
Symptoms n (%)	
Cough	6 (86)
Fever	2 (29)
Night Sweats	2 (29)
Weight Loss	2 (29)
Sputum Collected n (%)	7 (100)
GeneXpert Positive	7 (100)
Rifampin Resistant	1 (14)
AFB Smear Performed	7 (100)
Positive	5 (71)
Negative	2 (29)
Culture Reported	5 (71)
Positive	5 (100)

The 7 index cases attended 6 different schools (five primary schools and one secondary school), leading to the investigation of 1896 primary school and 119 secondary school students. No student refused to be screened for TB. The contact investigations took place a mean average of 221 days (range 70 to 325) after TB index case treatment initiation. Of the school contacts, 1754 (87%) were < 15 years of age and 1065 (53%) were female. 177 primary school students (10%) and 0 secondary school students screened positive for tuberculosis symptoms. Of this total, 132 children (75%) produced sputa on which a GeneXpert test was performed. No children had a positive GeneXpert result and none were clinically diagnosed with TB (Table 2A). All teachers were offered TB screening through school administrators; only 2% (6/243) accepted.

**Table 2:** Characteristics and yield of household vs. school contacts among index cases.

A) School contact investigations							
	School Type	School Contacts	FemaleGender	Age < 15 years	Positive TB Screen	Sputum Collected	GeneXpert Positive
IC 1	Primary	736	367	709	55	37	0
IC2, IC5	Primary^	461	269	448	33	17	0
IC 3	Primary	229	132	205	32	24	0
IC 4	Primary	244	127	232	15	13	0
IC 6	Secondary	119	72	0	0	0	0
IC 7	Primary	226	98	160	42	41	0
Total N		2015	1065	1754	177	132	0
*%*			*53*	*87*	*9*	75*	*0*
B) Household contact investigations							
	Home vs. facility screen	Household Contacts	FemaleGender	Age < 15 years	Positive TB Screen	Sputum Collected	GeneXpert Positive
IC 1	Facility	4	2	3	3	3	1
IC 2	Home	7	4	4	2	4	0
IC 3	Facility	5	2	2	0	1	0
IC 4	Home	6	3	1	5	2	0
IC 5	Facility	3	2	1	3	0	0
IC 6	Facility	7	6	0	7	5	0
IC 7	Home	8	6	4	4	4	0
Total N		40	25	15	24	19	1
%			*63*	*38*	*60*	79*	*5*

* percentage denominator equals total with positive TB symptom screens

^ denotes index cases that attended the same school

Investigation of the index case households led to the evaluation of 40 contacts. The evaluation of the household contacts took place a mean average of 25 days (range -8 to 114 days) after TB index case treatment initiation. Of the 7 households, the Butimba team evaluated 3 through home visits and 4 presented to the health facility. Of these household contacts, 15 (38%) were children < 15 years of age and 25 (63%) were female. 24 (60%) of household contacts reported one or more symptoms consistent with TB and 19 (79%) produced sputa for collection. One additional case of TB was identified by GeneXpert in these household contacts (Table 2B).

Contacts at school were less likely than household contacts to be positive by a standardized TB symptom screen (Odds Ratio (OR) 0.06, 95% Confidence Interval (95% CI) 0.03–0.12, P < 0.0001) but the data shows no difference in the likelihood of having sputum collected following a positive screen (OR 0.77, 95% CI 0.30–2.18, P = 0.80) (Table 3). The number of students needed to screen to identify an additional case of TB, following school contact with infectious TB, could not be calculated as no additional cases were identified. Among the 7 microbiologically confirmed index cases described here, the Butimba project screened 40 household contacts to identify one new TB case. The odds ratio among this cohort for developing TB disease following school vs. household TB exposure was 0.00 (95% CI 0.00 to 0.18, P = 0.02) (Table 3).

**Table 3:** Characteristics of contacts and outcomes associated with school vs. household TB exposure.

	Odds Ratio	95% CI	P value
TB Symptom Screen Positive	0.06	0.03–0.12	<0.0001
Sputum Collection Performed*	0.77	0.30–2.18	0.80
TB Disease	0	0–0.18	0.02

*Calculated from the total number of contacts with a positive symptoms screen

## Discussion

TB case finding activities are recommended—but not routinely implemented—in TB high-burden settings. The WHO recommends contact investigations among household contacts and close contacts of those with infectious TB[1,2]. However, perhaps due to the challenges of successfully implementing household contact investigations, limited work has been done to investigate the yield of contact investigations in close TB contacts outside of the home. Despite this, there is a growing body of evidence that after early childhood most TB infection occurs outside of the home environment in TB high burden settings. Adolescents are at high risk for incident TB infection[9], and as much as 50% of these infections occur in the school environment[11].

It is standard protocol—in high resource, TB low-burden settings—to investigate TB contacts in congregate settings outside of the household, such as school and work[14]. Older children (age 12–16) with smear positive disease and children 3–11 years, irrespective of smear status, transmit infection to 5–48% and 66–69% of close school contacts, respectively[15,16]. A recent study demonstrated transmission in 21% of close school contacts of older AFB smear positive children, but virtually no transmission in close contacts of smear negative or young children < 5[17]. Therefore, a risk of transmission exists following exposure to another student with TB, but the magnitude of risk may depend on a number of factors including index case age and smear status. Overall, in low TB burden, high-resource settings, investigation of school contacts represents an important opportunity to prevent disease in those identified with TB infection through tuberculin skin tests or interferon gamma release assays, but in most cases, few (if any) cases of TB disease are identified[15–17].

In contrast, Swaziland and other high-burden settings do not test typically for TB infection following exposure to an infectious TB case. In Swaziland, a symptom-based screening algorithm is recommended after exposure to determine the need for sputum-based testing by GeneXpert. Following close contact with an index case and after symptom-based exclusion of TB disease, isoniazid preventive therapy is recommended for contacts < 5 years of age or living with HIV. Immunologic tests for TB infection are not available. Although interferon gamma release assays demonstrate potential in such settings[18], screening for symptoms without testing for tuberculosis infection is the most cost-effective approach in managing household contacts[19]. In TB high-burden countries, the utility of school contact symptom screening with regard to secondary case identification is unknown. Therefore, our school contact investigations provide important new data.

In our population of 2015 school contacts symptom-screened for TB, we identified 0 cases of active TB. At the time, TB prevalence in Swaziland was estimated to be 733 cases per 100,000 individuals[20]. While likely lower among adolescents attending school, it was none-the-less surprising that no secondary cases or even independent incident cases were identified through this intervention. This is especially remarkable given all index cases were GeneXpert positive and the majority were smear positive. Two index cases attended the same school but the first case had rifampin resistance identified on GeneXpert and follow-up drug susceptibility testing, the second did not, suggesting this was not a linked case.

In comparison, a similar study from a high HIV/TB setting evaluated the prevalence of TB in an adolescent school-based population and found a TB prevalence of 3/1000 adolescent South African school students screened. This program performed screening irrespective of recent TB exposure and also evaluated several screening algorithms for sensitivity and specificity. Among 16 cases of culture-confirmed TB, 14 did not report any symptoms, suggesting that symptom-based screens may not have adequate sensitivity in high-risk adolescent populations[21].

The dependence on symptom screening for further testing in this study may have limited the sensitivity of the contact investigation. Compared to household contacts, a much lower percentage of school contacts screened positive for TB. This may be unique to adolescents, and/or may indicate the need to provide a more confidential environment for TB screening at schools. The symptom based screening algorithm may have limited case detection, though the approach kept with the programmatic recommendations for TB screening in Swaziland[22]. Screening algorithms—independent of symptom screens—may need to be evaluated further in this population, and the reliance on a symptom screen is a limitation of this analysis. Additionally, the limited evidence from both studies highlights the need to determine the most effective approaches for evaluating close child and adolescent TB contacts at schools in TB high-burden settings.

Other factors that may have also contributed to the low yield of school-based contact investigation include the possibility that children with TB symptoms may have been absent from school. Also, the screenings took place during the respiratory virus season, potentially decreasing the specificity of the screening questions. Additionally, screening of household contacts took place much sooner after identification of the index case, compared with school contacts (mean 25 vs. 203 days). Hence, it is possible that prior identification and treatment of secondary cases at schools occurred before the school interventions took place. Approximately 25% of school and household contacts with a positive screen did not provide sputum either due to reassessment of the need by the clinician, patient refusal or inability to produce an appropriate specimen. This may have also limited the sensitivity or our approach. In collaboration with the primary schools, the program was designed to evaluate all children in the grade of the index case and additionally all children in grades 5–7 in order to maintain index case confidentiality [14]. Focusing on those with the most contact, for example sharing the same classroom, may have decreased the resources required to perform the screening, but this focused approach would not have increased the yield of new cases.

It is also important to note that only 2% of teachers were willing to undergo TB screening and thus this group could not be evaluated as potential TB cases. This refusal also raises the issue of potential stigma related to TB and again may have biased the students against reporting symptoms. The SHP recommended providing a holistic health screening to teachers to include weight, BP and glucose monitoring, HIV testing services and TB screening. TB screening may have been more acceptable if provided as part of a larger bundle of services.

Household contact investigations of the same index cases in this study yielded one additional TB case among 40 contacts, suggesting that this is a more efficient approach to identify secondary TB cases. Additionally, screening of household contacts in the Butimba project as a whole yielded a new case of TB for every 63 persons screened and a new case of microbiologically confirmed TB for every 112 persons screened[12]. Without doubt, household contact investigations are a proven strategy to detect secondary TB cases and detect them earlier than otherwise identified through passive case finding[4,23]. Further, household contact investigations often identify the most vulnerable contacts, children < 5 years, affording the opportunity for early detection of disease before dissemination as well as prevention[24].

Our experience demonstrated the feasibility of symptom based school contact investigations, in a TB high-burden setting, but low-yield to identify secondary cases of TB. However, epidemiologic data from TB high-burden settings like this indicate that school is an important setting where adolescents obtain new TB infections. Therefore, school contact investigation has potential to be an important tool of TB control in TB high-burden settings. Nevertheless, the yield of these investigations can likely be increased if informed by further research defining new screening strategies that are not symptom dependent. Incorporating tests of TB infection or establishing a screening environment with more privacy from schoolmates may be important when performing contact investigations in this population. Programmatic resources for active case finding should remain focused on household contact investigations—a proven tool to prevent TB in high-risk children and identify new cases among close TB contacts.

## Supporting information

S1 Fig School Contact Tracing Tool. (DOCX)

S2 Fig Description of educational activities undertaken at school screening days. (DOCX)
