# Supplementary material for: School and household tuberculosis contact investigations in Swaziland: Active TB case finding in a high HIV/TB burden setting
Source: PLoS One. 2017 Jun 5;12(6):e0178873. doi: 10.1371/journal.pone.0178873 (PMC5459449; doi:10.1371/journal.pone.0178873)
Supplement: S1 Fig — (DOCX) [file pone.0178873.s001.docx]

***DEMOGRAPHIC INFORMATION***

CODE

**Date of Visit:**_____________ **School:** _________________________________ **School Location:** _______________________________________ **Cough Monitor:** ___________________________________

**☐ Teacher** / **☐ Student / ☐ Other:______________**_______ **☐ Classmates:** *Grade/Form (Number):* ________ *Class (Letter):*_________ **☐ Boarding Student *(****Dorm:* ______________Room:_______________)

***INDEX CASE INFORMATION (To be completed after school visit):* First Name:** ________________________ **Surname:** _____________________ **TB Registration Number:** ___________________________ **BMU:** __________

| **Contact’s Name** | **DOB** | **Gender**  (M / F) | **HIV Status**  (NR, R, Unk*)* | **TB Screen**  (C – Cough, F – Fever,  NS – Night Sweats,  WL – Weight Loss)  *Circle if Present.* | **Sputum Collected?**  (S – School, F – Facility,  H – Home)  **Do not collect sputum if child is on TB medication.** | **Caregiver’s Name & Phone Number**  *(Only if positive TB screen.)* | ***If boarding student:***  **Sleeping Location** | **Results** |
| --- | --- | --- | --- | --- | --- | --- | --- | --- |
|  |  |  |  | ☐ Negative  ☐ Positive  C F NS WL | ☐ No ☐ Yes  S F H  Date if collected in the F or H:  / / |  | Dorm:  Room:  Adj Bed | **GXP:** ☐ MTB ND ☐Invalid/Indet ☐Rejected ☐MTB Det *(Rif: ☐ R ☐ ND)*  **SMEAR:** ☐Neg ☐Scanty ☐1+ ☐2+ ☐3+  **CXR:** ☐ Normal ☐ Positive (Date:_______________)  **ATT Initiation Date:** ____________ **Facility:**_________________  **CULTURE:** ☐Neg ☐POSITIVE Rif *☐ S ☐ R* INH *☐ S ☐ R* |
|  |  |  |  | ☐ Negative  ☐ Positive  C F NS WL | ☐ No ☐ Yes  S F H  Date if collected in the F or H:  / / |  | Dorm:  Room:  Adj Bed | **GXP:** ☐ MTB ND ☐Invalid/Indet ☐Rejected ☐MTB Det *(Rif: ☐ R ☐ ND)*  **SMEAR:** ☐Neg ☐Scanty ☐1+ ☐2+ ☐3+  **CXR:** ☐ Normal ☐ Positive (Date:_______________)  **ATT Initiation Date:** ____________ **Facility:**_________________  **CULTURE:** ☐Neg ☐POSITIVE Rif *☐ S ☐ R* INH *☐ S ☐ R* |
|  |  |  |  | ☐ Negative  ☐ Positive  C F NS WL | ☐ No ☐ Yes  S F H  Date if collected in the F or H:  / / |  | Dorm:  Room:  Adj Bed | **GXP:** ☐ MTB ND ☐Invalid/Indet ☐Rejected ☐MTB Det *(Rif: ☐ R ☐ ND)*  **SMEAR:** ☐Neg ☐Scanty ☐1+ ☐2+ ☐3+  **CXR:** ☐ Normal ☐ Positive (Date:_______________)  **ATT Initiation Date:** ____________ **Facility:**_________________  **CULTURE:** ☐Neg ☐POSITIVE Rif *☐ S ☐ R* INH *☐ S ☐ R* |
|  |  |  |  | ☐ Negative  ☐ Positive  C F NS WL | ☐ No ☐ Yes  S F H  Date if collected in the F or H:  / / |  | Dorm:  Room:  Adj Bed | **GXP:** ☐ MTB ND ☐Invalid/Indet ☐Rejected ☐MTB Det *(Rif: ☐ R ☐ ND)*  **SMEAR:** ☐Neg ☐Scanty ☐1+ ☐2+ ☐3+  **CXR:** ☐ Normal ☐ Positive (Date:_______________)  **ATT Initiation Date:** ____________ **Facility:**_________________  **CULTURE:** ☐Neg ☐POSITIVE Rif *☐ S ☐ R* INH *☐ S ☐ R* |
|  |  |  |  | ☐ Negative  ☐ Positive  C F NS WL | ☐ No ☐ Yes  S F H  Date if collected in the F or H:  / / |  | Dorm:  Room:  Adj Bed | **GXP:** ☐ MTB ND ☐Invalid/Indet ☐Rejected ☐MTB Det *(Rif: ☐ R ☐ ND)*  **SMEAR:** ☐Neg ☐Scanty ☐1+ ☐2+ ☐3+  **CXR:** ☐ Normal ☐ Positive (Date:_______________)  **ATT Initiation Date:** ____________ **Facility:**_________________  **CULTURE:** ☐Neg ☐POSITIVE Rif *☐ S ☐ R* INH *☐ S ☐ R* |
