# Supplementary material for: School and household tuberculosis contact investigations in Swaziland: Active TB case finding in a high HIV/TB burden setting
Source: PLoS One. 2017 Jun 5;12(6):e0178873. doi: 10.1371/journal.pone.0178873 (PMC5459449; doi:10.1371/journal.pone.0178873)
Supplement: S2 Fig — (DOCX) [file pone.0178873.s002.docx]

**Supplemental Figure 2: Educational activity description**


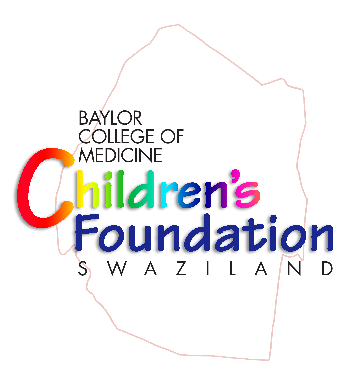


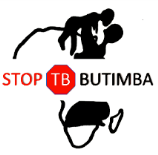


**Educational Activity during School Contact Tracing Program**

| **Time** | **Activity** | **Persons Responsible** | **Description** |
| --- | --- | --- | --- |
| 15 minutes | Explanation of Event and Health Talk | School Health Program Nurses | School Health Programme describes the screening process and the causes, risks, and symptoms of TB. School Health Programme also discusses the different between latent and active TB disease. |
| 15 – 20 minutes | Activity | Baylor Staff | Kick TB Risk Field – students dribble a soccer ball in two teams through risk factors and symptoms of TB. |
|  | Screening | Baylor Staff, School Health Program | Students are screened for TB and given take-home screening tool |

**Kick TB Risk Field Activity**

**Goals:**

- Participants will be able to explain 3 risks for getting TB disease

**Materials:**

- at least 10 soccer cones or obstacles
- at least 2 soccer balls
- risk cards
- playing field

**Preparation:**

- Decide how many teams you will play TB Risks with based on the size of the group and the amount of cones/soccer balls available
- Make the appropriate number of lines/rows and attach the risk cards to the cones using sticks and cellotape

**TB Risk Field Activity Directions:**

1. Introduce TB Risks

- **Question: What is a risk? Answer:** Something that can put us in danger
- **Question: What are some risks for getting TB? Answer:** weakened immune system, substance abuse, closed/unventilated spaces, poor nutrition, crowded spaces, not taking preventative medication , TB contacts
- **In Risk Field, we are going to be talking about these risks.**
- **Key Message: There are many risks in life that can lead to TB infection.**

2. Students dribble a soccer ball in two teams through “risks” and “symptoms” (set up as cones labeled: Closed Windows, Coughing, Fevers, Not Taking Medication, etc.) of catching TB. They then try to score a goal and pass the ball to the next person on their team. Each person on the team continues until one team finishes before the other.
